# High ankle-brachial index predicts cardiovascular events and mortality in hemodialysis patients with severe secondary hyperparathyroidism

**DOI:** 10.1590/2175-8239-JBN-2020-0218

**Published:** 2021-05-12

**Authors:** Alinie Pichone, Gabriela Campos, Maurilo Leite, Carlos Perez Gomes

**Affiliations:** 1 Universidade Federal do Rio de Janeiro, Hospital Universitário Clementino Fraga Filho, Divisão de Nefrologia, Rio de Janeiro, RJ, Brasil.

**Keywords:** Ankle Brachial Index, Cardiovascular event, Renal Dialysis, Hyperparathyroidism, Secondary, Vascular Calcification, Índice Tornozelo-Braço, Evento Cardiovascular, Diálise Renal, Hiperparatireoidismo Secundário, Calcificação Vascular

## Abstract

**Introduction:**

Vascular calcification related to severe secondary hyperparathyroidism (SHPT) is an important cause of cardiovascular and bone complications, leading to high morbidity and mortality in patients with chronic kidney disease (CKD) undergoing hemodialysis (HD). The present study aimed to analyze whether ankle-brachial index (ABI), a non-invasive diagnostic tool, is able to predict cardiovascular outcomes in this population.

**Methods:**

We selected 88 adult patients on HD for at least 6 months, with serum iPTH>1,000pg/mL. We collected clinical data, biochemical and hormonal parameters, and ABI (sonar-Doppler). Calcification was assessed by lateral radiography of the abdomen and by simple vascular calcification score (SVCS). This cohort was monitored prospectively between 2012 and 2019 for cardiovascular outcomes (death, myocardial infarction (MI), stroke, and calciphylaxis) to estimate the accuracy of ABI in this setting.

**Results:**

The baseline values were: iPTH: 1770±689pg/mL, P: 5.8±1.2 mg/dL, corrected Ca: 9.7±0.8mg/dL, 25(OH)vit D: 25.1±10.9ng/mL. Sixty-five percent of patients had ABI>1.3 (ranging from 0.6 to 3.2); 66% had SVCS≥3, and 45% aortic calcification (Kauppila≥8). The prospective evaluation (51.6±24.0 months), provided the following cardiovascular outcomes: 11% of deaths, 17% of nonfatal MI, one stroke, and 3% of calciphylaxis. After adjustments, patients with ABI≥1.6 had 8.9-fold higher risk of cardiovascular events (p=0.035), and ABI≥1.8 had 12.2-fold higher risk of cardiovascular mortality (p=0.019).

**Conclusion:**

The presence of vascular calcifications and arterial stiffness was highly prevalent in our population. We suggest that ABI, a simple and cost-effective diagnostic tool, could be used at an outpatient basis to predict cardiovascular events in patients with severe SHPT undergoing HD.

## Resumo

**Introdução:**

A calcificação vascular relacionada ao hiperparatireoidismo secundário (HPTS) grave é uma causa importante de complicações cardiovasculares e ósseas, levando a alta morbidade e mortalidade em pacientes com doença renal crônica (DRC) em hemodiálise (HD). O presente estudo objetivou analisar se o índice tornozelo-braquial (ITB), uma ferramenta diagnóstica não invasiva, pode predizer desfechos cardiovasculares nesta população.

**Métodos:**

Selecionamos 88 adultos em HD há pelo menos 6 meses, com PTHi sérico>1.000pg/mL. Coletamos dados clínicos, parâmetros bioquímicos e hormonais, e ITB (sonar-Doppler). A calcificação foi avaliada por radiografia lateral do abdome e por escore de calcificação vascular simples (ECVS). Esta coorte foi monitorada prospectivamente entre 2012 e 2019 para desfechos cardiovasculares (óbito, infarto do miocárdio (IM), acidente vascular cerebral e calcifilaxia) para estimar a precisão do ITB neste cenário.

**Resultados:**

Os valores basais foram: PTHi: 1770±689pg/mL, P: 5,8±1,2 mg/dL, Ca corrigido: 9,7±0,8mg/dL, 25(OH)vit D: 25,1±10,9ng/Ml; 65% dos pacientes apresentaram ITB>1,3 (variando de 0,6 a 3,2); 66% tiveram ECVS≥3, e 45% calcificação da aorta (Kauppila≥8). A avaliação prospectiva (51,6±24,0 meses) forneceu os seguintes desfechos cardiovasculares: 11% de óbitos, 17% de IM não fatal, um AVC, 3% de calcifilaxia. Após ajustes, pacientes com ITB≥1,6 tiveram risco 8,9 vezes maior de eventos cardiovasculares (p=0,035), e ITB≥1,8 apresentaram risco 12,2 vezes maior de mortalidade cardiovascular (p=0,019).

**Conclusão:**

A presença de calcificações vasculares e rigidez arterial foi altamente prevalente em nossa população. Sugerimos o ITB, uma ferramenta diagnóstica simples e econômica, para ser usada em ambulatório para prever eventos cardiovasculares em pacientes com HPTS grave em HD.

## Introduction

Vascular calcification is a strong prognostic marker of total and cardiovascular mortality in hemodialysis patients^1^. The occurrence of this process can be both passive and active, with the presence of calcification of atherosclerotic plaques, injury to the vessel wall due to uremia, oxidative stress or inflammation, and depends on the balance between inductors (bone sialoprotein, alkaline phosphatase, osteocalcin, osteonectin, BMP-2) and inhibitors of vascular calcification (matrix GLA protein, osteoprotegerin, fetuin-A, pyrophosphate, BMP-7)^2^^-^ 4.

Vascular smooth muscle cells can differentiate into osteoblast-like cells, which produce proteins, collagen, and alkaline phosphatase and predispose to deposition of hydroxyapatite in vascular wall, leading to vascular stiffness^2^. This active process of phenotypic change is triggered by the activation of the core binding factor α-1 (Cbfa-1) promoted by phosphorus. The accumulation of phosphorus with the progression of chronic kidney disease (CKD) is directly involved in the pathogenesis of secondary hyperparathyroidism (SHPT)^5^. In addition, severe SHPT increases bone turnover, which further elevates phosphatemia by increased bone resorption due to osteoclastic activity^6^. Both factors contribute to progression of vascular calcification.

In addition to hyperphosphatemia, high PTH is associated with several other alterations such as calcium disturbance, increase of CaxP product, low levels of vitamin D, and anemia. These factors may be responsible for the increased morbidity and mortality found in patients with severe SHPT^7^. The deposit of calcium in the vessel wall can be detected by radiographic examination. Adragão et al. suggested a semiquantitative simple vascular calcification score (SVSC) using pelvis and hand radiographs^8^ and Kauppila score detects lumbar aortic calcification^9^. All these methods are well known predictors of cardiovascular events in dialysis population^8^^,^ 9. Furthermore, the ankle-brachial index (ABI), a simple and inexpensive tool that can be performed on physical examination, can also be applied in order to evaluate vessel stiffness. Obstructive arterial disease can be identified by low ABI (<0.9), and high ABI (>1.3) has been associated with vascular calcification due to arterial incompressibility^10^^,^ 11. In dialysis patients, low and high ABI were related to cardiovascular events and mortality^10^^,^ 12 ^-^ 14.

The aim of this study was to investigate the role of ABI on the prediction of cardiovascular outcomes in hemodialysis patients with severe SHPT.

## Materials and Methods

This prospective, observational, single-center study evaluated 88 patients from the outpatient clinic for mineral bone disorder of chronic kidney disease (CKD-MBD) at the University Hospital Clementino Fraga Filho from Federal University of Rio de Janeiro (UFRJ). The protocol was approved by the local ethics committee, in accordance with the Declaration of Helsinki. We included patients older than 18 years, under hemodialysis for at least 12 months, who had iPTH>1000pg/mL. We excluded patients with known bone disease (Paget, multiple myeloma, metastases) and using medications that affect bone metabolism or vascular calcification (anticonvulsants, warfarin, and corticosteroids). Patients with less than 12 months of follow-up were also excluded.

We analyzed clinical and laboratorial data, radiographs, and ABI measurement at baseline. In relation to laboratory tests, we evaluated serum total calcium (8.5-10.5mg/dL), phosphorus (2.5-4.5mg/dL), alkaline phosphatase (65-300U/L), iPTH (second generation chemiluminescent immunometric assay, 12-65pg/mL), 25(OH) vitamin D (30ng/mL), albumin (3.6-4.8g/dL), C-reactive protein (0-5mg/dL), ferritin (28-365ng/mL), transferrin saturation index (20-50%), hemoglobin (12-16g/dL), and bicarbonate (22-26mmol/L). ABI was determined using adequate sphygmomanometer for arm and leg circumferences and sonar-Doppler Medpej DF7001VN, 10MHz probe. The examination was performed in the interdialytic period with the patient in supine position with the extremities at the same level as the heart, after 10 minutes of rest. We measured arterial pressure on the arm without arteriovenous fistula and in both lower limbs. The ABI was calculated by dividing the ankle systolic blood pressure (SBP) / brachial SBP. Values ​​below 0.9 are associated with peripheral arterial disease, between 0.9 and 1.3 are normal, and values greater than 1.3 are associated with vascular calcification and vessel incompressibility^11^.

The radiographs were analyzed by two authors and blinded for the name, biochemical results, and ABI value of patients. We evaluated the SVCS (Adragão score) by hand and pelvis radiographs. Each film was divided into 4 quadrants and only linear calcifications were considered in vessel topography. The presence of calcification in each quadrant counted 1 point, with the total score ranging from 0 to 8 points^8^. Aortic calcification was analyzed by the lateral radiograph of the abdomen and we calculated the abdominal aortic calcification (AAC) score developed by Kauppila^9^, which evaluates calcification in anterior and posterior wall of the aorta over the first to fourth lumbar vertebra (L1-L4); calcification length smaller than 1/3 of vertebra was given 1 point, smaller than 2/3 was given 2, and higher than 2/3 was given 3 points, with total score raging from 0 to 24.

The study population was prospectively monitored in an outpatient basis between April 2012 and October 2019. Cardiovascular outcomes were death, cardiac arrhythmia (documented by electrocardiographic study), acute myocardial infarction (confirmed by cardiac catheterization), stroke (diagnosed by brain computed tomography), or calciphylaxis (confirmed by radiological and histopathological exams).

Statistical analysis was performed using SPSS v24. Data are expressed as percentages or mean ± standard deviation. Unpaired Student's t-tests (continuous variables) and Chi-square tests (categorical variables) were used for comparison between two independent groups. The cutoff level of ABI was calculated by Receiver Operating Characteristic (ROC) curve, and Kaplan-Meier survival analysis with Log rank test, followed by Cox Regression Model for prediction of hazards and risks. P-value <0.05 was considered statistically significant.

## Results

Of the 88 patients, 46% were male and 50% had white skin color, with mean age of 47.4±10.8 years, and on hemodialysis for almost ten years. The main clinical and laboratory characteristics are shown in Table 1. Hypertension was the main cause of renal disease, in 23% the cause was undetermined, and only 2 patients had diabetes mellitus. The patients were on intermittent hemodialysis (three sessions a week), with dialysate calcium concentration of 3.0mEq/L. At admission, 9% had history of fracture, 100% of patients were in use of phosphorus binders (sevelamer and/or calcium carbonate), 61% in use of vitamin D analogues (12% used cholecalciferol, 57% calcitriol, and 3% paricalcitol), and 36% in use of cinacalcet.

**Table 1:** Clinical and biochemical parameters of total population

Parameters (N=88)	Mean ± SD or N (%)
Age (years)	47.4±10.8
Gender (M)	40 (46%)
BMI (kg/m^2^)	24.1±4.8
HD vintage (months)	117±54.2
Causes of CKD	
Hypertension	32 (36%)
Undetermined	20 (23%)
Chronic glomerulonephritis	6 (7%)
APKD	5 (6%)
Chronic pyelonephritis	5 (6%)
Diabetes	2 (2%)
Ca (mg/dL)	9.7±0.8
P (mg/dL)	5.8±1.2
iPTH (pg/mL)	1770±688.9
25OHVitD (ng/mL)	25.1±10.9
ALP (UI/L)	1466.9±1156.2
Alb (g/dL)	3.8±0.5
Hb (g/dL)	10.9±2.1
HCO3 (mmol/L)	21.7±5.4
Mg (mg/dL)	2.2±0.5
TSAT (%)	28.2±13.6
Ferritin (ng/mL)	903.9±872.7
CRP (mg/L)	25.6±31.5
ABI	1.8±0.7

BMI: body mass index; APKD: autosomal dominant polycystic kidney disease; HD: hemodialysis; ABI: ankle-brachial index; iPTH: parathyroid hormone; ALP: alkaline phosphatase; Alb: albumin; Hb: hemoglobin; TSAT: transferrin saturation index; CRP: C-reactive protein.

All patients were on parathyroidectomy list, but only 50% did the surgical procedure. Eleven percent died before the procedure and 16% presented coronary disease or cardiac dysfunction that contraindicated the surgery. During the follow up period, all patients were treated with medications (vitamin D analogues, calcimimetic, phosphate binder, or calcium replacement) according to laboratory results, while they were waiting for parathyroidectomy. It should be noted that the use of calcitriol decreased, while calcimimetic and selective vitamin D analog increased significantly during follow-up.

The mean ABI at baseline was 1.8±0.7 (ranging from 0.6 to 3.2) and only five patients presented an ABI<0.9. Sixty-five percent of the patients presented ABI>1.3. In the evaluation of vascular calcification score based on plain radiographic films of pelvis and hand (SVCS or Adragão Score), 66% of the patients presented SVCS score ≥ 3. In the evaluation of vascular calcification score based on lumbar aortic calcification by X-ray (AAC or Kauppila score), 45% of the patients presented AAC score ≥ 8.

At follow up evaluation (51.6±24.0 months), we observed: 11% of deaths, 17% of nonfatal MI, one stroke, and 3% of calciphylaxis. As causes of deaths, one was sudden death and the others was fatal MI.

**Figure 1:**
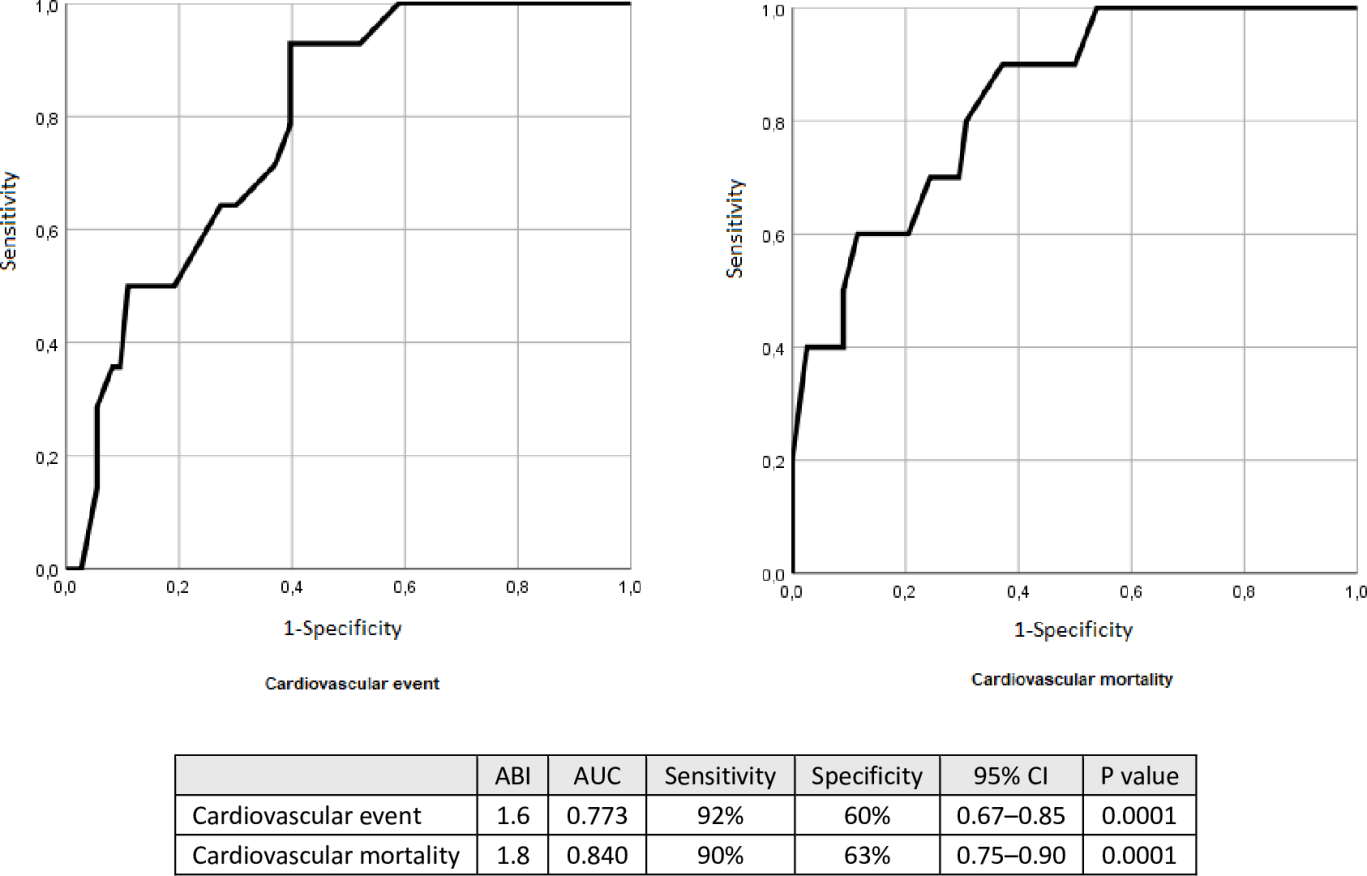
Receiver operating characteristic (ROC) curve analysis of ankle-brachial index (ABI) for cardiovascular event (LEFT) and cardiovascular mortality (RIGHT).

**Figure 2:**
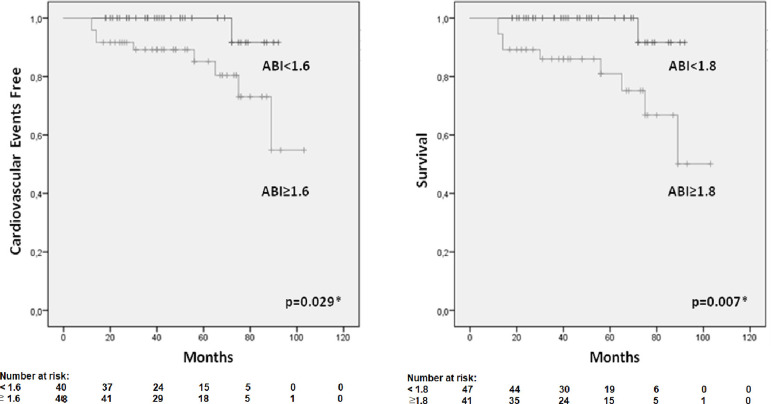
Kaplan-Meyer curves for occurrence of cardiovascular events (LEFT) comparing the ankle-brachial index (ABI) below 1.6 group versus ABU above 1.6 group and cardiovascular mortality (RIGHT) comparing the ABI below 1.8 group versus ABI above 1.8 group. *Log Rank (Mantel Cox).

We used the ROC curve to find the most accurate ABI value to predict cardiovascular events and mortality. A 1.6 ABI presented AUC of 0.773 (95%CI 0.67-0.85, p=0.0001), with 92% sensitivity and 60% specificity to predict cardiovascular events while an ABI of 1.8 presented AUC of 0.840 (95%CI 0.75-0.90, p=0.0001), with 90% sensitivity and 63% specificity to predict cardiovascular mortality (Figure 1). Kaplan-Meier curves were applied to evaluate the occurrence of events, comparing the groups ABI≥1.6 versus ABI<1.6 or ABI≥1.8 versus ABI<1.8 (Figure 2). The ABI≥1.6 group (p=0.029) had significantly more cardiovascular events, and patients of the ABI≥1.8 group (p=0.007) had significantly more cardiovascular deaths. In Cox Regression Model, the hazard ratio for the cardiovascular events was 9.7 (95%CI 1.26-75.0; p=0.03) in the ABI≥1.6 group. The hazard ratio for cardiovascular mortality was 9.9 (95%CI 1.25-78.4; p=0.03) in the ABI≥1.8 group. After adjustment for age and gender, the hazard ratio for cardiovascular events was 8.9 (95%CI 1.17-69.0; p=0.035) in the ABI≥1.6 group, and cardiovascular mortality was 12.2 (95%CI 1.51-99.7; p=0.019) in the ABI≥1.8 subgroup.

Based on these significant differences in cardiovascular outcomes, we analyzed the clinical, laboratory, and imaging characteristics of each group classified by ABI. As can be shown in Table 2, patients with ABI≥1.8 presented higher phosphatemia and HD vintage, while both groups had higher alkaline phosphatase and more vascular calcification (SVCS≥3 and AAC score≥8).

**Table 2:** Baseline characteristics of study participants according to abi (ankle-brachial index)

	ITB < 1,6 (n=40)	ITB ≥ 1,6 (n=48)	Valor de p*	ITB < 1,8 (n=47)	ITB ≥ 1,8 (n=41)	Valor de p*
Age (years)	46 .3±11 .8	48,3±9,9	0,401	46,6±11,6	48,3±9,8	0,453
HD vintage (months)	108 .3±57 .7	124,3±50,5	0,168	104,7±55,3	131,2±49,9	**0,021**
BMI (Kg/m2)	24 .0±4 .9	24,2±4,8	0,876	24,3±5,0	24,0±4,5	0,751
Ca (mg/dL)	9 .6±0 .9	9,7±0,8	0,624	9,6±0,9	9,7±0,8	0,533
P (mg/dL)	5 .5±1 .1	6,0±1,3	0,085	5,4±1,1	6,2±1,3	**0,004**
Mg (mg/dL)	2 .1±0 .6	2,3±0,4	0,276	2,2±0,5	2,3±0,4	0,484
25OH vit D (ng/mL)	29 .3±13 .9	25,8±7,4	0,143	29,0±13,1	25,5±7,4	0,144
ALP (U/L)	1196 .3±953 .9	1692,4±1266,7	**0,044**	1232,5±926,2	1735,6±1334,8	**0,041**
iPTH (pg/mL)	1774 .9±784 .7	1765,9±606,2	0,952	1750,3±747,4	1792,5±623,5	0,776
Albumin (g/dL)	3 .9±0 .5	3,9±0,5	0,949	3,9±0,5	3,8±0,5	0,648
HCO3 (mmol/L)	22 .0±5 .3	20,9±6,4	0,438	22,1±5,2	20,6±6,5	0,267
TSAT (%)	30 .2±13 .2	26,6±13,9	0,239	29,4±12,6	26,8±14,8	0,384
Ferritin (ng/mL)	902 .1±863 .2	905,3±889,4	0,987	943,7±867,2	860,2±887,6	0,664
CRP(mg/L)	20 .5±24 .7	29,9±35,9	0,178	21,4±23,5	30,5±38,5	0,188
Hb (g/dL)	11 .0±1 .9	10,8±2,2	0,620	10,9±1,9	10,9±2,3	0,888
SVCS≥3	10(26%)	47(98%)	**<0,001**	17(36%)	41(100%)	**<0,001**
AAC≥8	5(13%)	34(71%)	**<0,001**	6(13%)	33(81%)	**<0,001**

HD: hemodiálise; IMC: índice de massa corporal; FA: fosfatase alcalina; PTHi: paratormonio; IST: índice de saturação da transferrina; PCR: proteína C reativa; Hb: hemoglobina; ECVS: escore de calcificação vascular simples (Adragão); CAA: escore de calcificação da aorta abdominal (Kauppila).

* Teste T ou Qui-quadrado.

## Discussion

Despite several medications being available for the treatment of secondary hyperparathyroidism, this condition is highly prevalent, especially in developing countries where its severity is greater. According to DOPPS II study, 26.3% of patients present iPTH>300pg/mL^15^, while this prevalence reaches 51.4% in Argentina^16^. In South America there is a high prevalence of severe hyperparathyroidism (iPTH>1000pg/mL), reaching 13,3% in Argentina and 10.7% in Brazil^17^. Regarding biochemical findings, the last Brazilian census of dialysis^18^, as well as data from Europe, United States, and Japan^19^^,^ 20, all have consistently documented high prevalence of mineral and hormonal disorders, although these documents do not provide PTH subanalysis. As a comparison, our patients with severe SHPT (iPTH>1,000pg/mL) presented more hyperphosphatemia and hypovitaminosis D. High levels of CRP and ferritin found in our population strongly suggest chronic inflammation. The association of inflammation and metabolic acidosis also plays a role on the bone by reducing osteoblastic activity, osteoclastic increase and worsening SHPT, as it increases the release of PTH, the number of PTH receptors, and the activity of this hormone in its receptors, increasing cardiovascular and bone complications^21^.

In our study, the HD vintage was a risk for the development of severe SHPT, requiring parathyroidectomy^22^. Among the main causes of ESRD, hypertension was the most prevalent in our study. However, despite the fact that diabetes mellitus (DM) is the second cause of renal dysfunction in Brazil^18^, it had a low prevalence in this study. This may be due to diabetic patients presenting 20-50% lower levels of PTH and low bone remodeling, either by hyperglycemia (accumulation of glycation end-products), reduction of insulin growth factor-1 (which stimulates the proliferation of osteoblasts), increased sclerostin, or hormonal changes (gonadal dysfunction)^23^.

The presence of vascular calcification is associated with a high risk for cardiovascular event and mortality^1^. The stiffness of the vessels increases the afterload, leading to left ventricular hypertrophy (LVH), increased myocardial demand, and subsequent diastolic and systolic dysfunction. In addition, coronary disease and reduced coronary perfusion by LVH increase the risk of acute myocardial infarction, cardiac dysfunction, arrhythmias, and heart failure^24^^-^ 26. Calciphylaxis or calcific uremic arteriolopathy (CUA) is a severe calcification of small vessels, such as dermis and subcutaneous arterioles, and can also determine reductions in the arteriolar blood flow and serious complications, leading to high mortality and morbidity^27^.

For the measurement of ABI, we used the method with the best accuracy (sonar-Doppler). According to the American Heart Association, normal values ​​for ABI are between 0.9 and 1.3; an ABI≥1.4 is related to vascular incompressibility due to vessel calcification. It should be emphasized that the presence of stiff vessels do not exclude occlusive lesions, but the stenotic disease cannot be detected by ABI^11^. In our study, the majority of the patients presented vascular stiffness according to ABI, probably due to the severity of SHPT and high prevalence of hyperphosphatemia. Despite several studies in which an ABI>1.3 demonstrated a higher occurrence of cardiovascular events, in patients either on dialysis^10^^,^ 12 ^-^ 14 or not^28^^-^ 32, we did not find studies in patients with severe SHPT. London et al.^33^ demonstrated that low bone turnover was related to peripheral arterial disease (PAD) and it was inversely related to parathyroidectomy (PTX). Furthermore, that study showed that more than 54% of patients with ABI>1.4 had been submitted to PTX, and this fact may suggest the increase of vascular calcification in patients who presented elevated PTH.

It is well known that ABI<0.9 is associated with PAD and that decreasing values ​​are related to the severity of disease, as patients with ABI<0.4 present severe PAD^34^. However, the same does not happen with high ABI, since it is known to be related to the incompressibility of the vessel, but there is no degree of severity. As the prevalence of ABI>1.3 in our study was very high, we evaluated if patients with higher ABI values presented more outcomes. The ROC curve defined the value of ABI≥1.6 as the best predictor of cardiovascular event and ABI≥1.8 a predictor of cardiovascular mortality. These findings suggest that the greater the stiffness of the vessels, the greater the severity of the cardiovascular outcome.

In 2004, Adragão and colleagues assessed patients on dialysis, regardless of PTH level, and suggested a simple method for assessing vascular calcification by the presence of calcium deposit in the vessels in each quadrant in the hand and pelvis radiographs, with the maximum of 8 points^8^. These authors demonstrated the association of this score with cardiovascular events when the SVCS was ≥3. The observation of linear calcium deposits in small vessels, as in the hand, a site usually resistant to atherosclerosis, indicate a medium calcification^3^ and consequently greater vascular stiffness. In our study, almost all patients with ABI≥1.6 had SVCS≥3 and AAC score≥8. By using sonar-Doppler, the incompressibility caused by calcium deposits in the vascular wall, visualized in aortic, pelvis, and hands radiographs, could be examined at bedside. We also found a strong positive correlation between ABI and SVCS, in agreement with Adragão et al.^10^. They demonstrated that an ABI>1.3 was associated with peripheral and distal arteries calcification and an increased risk of cardiovascular and total mortality of patients.

When we analyzed the groups according to ABI, both ABI≥1.6 and ABI≥1.8 groups presented higher alkaline phosphatase. The elevation of this marker of osteoblastic activity may reflect the increase in bone turnover promoted by high PTH, which stimulates both bone formation and bone resorption and may be associated with increased phosphorus and calcium efflux to the blood^35^. Fractures can also increase the levels of this biomarker^36^, but in our study there was no difference in patients with fractures between groups. However, studies have already shown that the increase of tissue nonspecific alkaline phosphatase may be related to vascular calcification since pyrophosphate, inhibitor of vascular calcification, can be degraded by tissue phosphate alkaline phosphatase, thus contributing to hydroxyapatite deposition and, consequently, arterial calcification^3^^,^ 4 ^,^ 37.

We also observed that hyperphosphatemia presented a significant difference in group ABI≥1.8, suggesting the role of phosphorus for the pathophysiology of vascular calcification^3^, increasing Runx-2 expression, and differentiation of vascular smooth muscle cells into osteoblast-like cells. The NEFRONA study^38^ demonstrated that an ABI>1.4 is related to HD and hyperphosphatemia. Several authors found a positive correlation between serum phosphorus and vascular calcification, being both variables also associated with cardiovascular events^(1, 39,40)^. The association of elevated ABI with HD vintage found in this study has also been observed by other authors^33^^,^ 40, suggesting that patients exposed to mineral disturbances for long period of time could develop more stiffness.

Our study population was composed of patients with very severe hyperparathyroidism and consequent bone and extra-skeletal disorders. The prevalence of vascular calcification in these patients was quite high and detected by a widely available and low-cost tool such as the sonar-Doppler. To our knowledge, this was the first study to evaluate ABI as predictor of cardiovascular outcome in hemodialysis patients with severe hyperparathyroidism. We showed that patients with ABI≥1.6 presented a nine-fold increased risk of presenting a cardiovascular event, while patients with ABI≥1.8 had twelve-fold higher risk of cardiovascular death. This information should be considered when choosing a therapy. As stated earlier, serious SHPT is a reality in many countries around the world, either due to the difficulty in acquiring the medications or performing parathyroidectomy in specialized centers. Furthermore, future studies may evaluate whether higher ABI levels may represent different degrees of severity, as already occurs with low ABI levels.

The main limitation of the present study was the observational single center design, with a relatively small number of patients. Furthermore, we used only the laboratory tests available in clinical practice and we were not able to evaluate biomarkers, such as FGF-23 or fetuin A, which would provide more information about the pathophysiology of vascular calcification.

In conclusion, the present study showed that the use of ABI by sonar-Doppler may provide better knowledge about prevalence of vascular calcifications in severe SHPT, as well as clinical outcomes. We suggest that this simple diagnostic tool be used as predictor of cardiovascular events and mortality, especially on patients with ABI≥1.6 in HD patients with severe SHPT. This easy and relatively cost-effective tool may add on therapeutic decision and help select those patients that should be prioritized for parathyroidectomy.
